# Addressing gender disparities in the efficacy of psychological interventions for behavioral addictions: protocol for a systematic review and individual patient data meta-analysis of randomized controlled trials

**DOI:** 10.1186/s13722-026-00668-0

**Published:** 2026-05-22

**Authors:** Nora M. Laskowski, Hannah R. Hambruch, Christopher Zaiser, Isabel Schnorr, Daniel Maier, Katharina S. Appel, Jörg Janne Vehreschild, Georgios Paslakis

**Affiliations:** 1 University Clinic for Psychosomatic Medicine and Psychotherapy, Medical Faculty, Campus East-Westphalia, Ruhr-University Bochum, Virchowstr. 65, 32312 Luebbecke, Germany; 2 Faculty of Medicine, Institute for Digital Medicine and Clinical Data Sciences, Goethe University Frankfurt, Marienburgstr. 6, 60528 Frankfurt am Main, Germany; 3 German Cancer Consortium (Deutsches Konsortium für Translationale Krebsforschung; DKTK), Partner Site Frankfurt/Mainz, a partnership between German Cancer Research Center (Deutsche Krebsforschungszentrum; DKFZ) and University Medicine Frankfurt, Frankfurt am Main, Germany

**Keywords:** Behavioral addictions, Psychological interventions, Treatment efficacy, Gender differences, Individual patient data meta-analysis

## Abstract

**Background:**

Behavioral addictions (BAs) are increasingly recognized as significant mental health challenges, but evidence on treatment efficacy remains limited. Psychological interventions, particularly cognitive-behavioral therapy (CBT) and its internet-based formats, are considered first-line treatments. However, it remains unclear whether these interventions are equally effective across genders. Addressing this gap is essential for developing gender-sensitive treatment strategies and improving clinical outcomes in individuals with BAs.

**Methods:**

This systematic review and individual patient data meta-analysis (IPDMA) will evaluate the efficacy of psychological interventions for BAs and examine gender as a potential moderator of intervention outcomes. The primary endpoint is the change in BA symptom severity from baseline to end of treatment, with treatment × gender interaction effects estimated. Eligible randomized controlled trials (RCTs) will be identified through comprehensive database and trial register searches. Study selection, data extraction, and risk-of-bias (RoB) assessment will be conducted independently by two reviewers. RoB will be assessed using the Cochrane RoB 2 tool, and certainty of evidence will be evaluated using the GRADE framework. Principal investigators will be contacted to provide anonymized patient-level data. Data will be synthesized using a two-stage IPDMA with random-effects models, allowing inclusion of trials providing either individual-level data or interaction estimates. Statistical heterogeneity will be quantified using the I^2^ statistic, and exploratory analyses will examine intervention characteristics and BA subtypes. Screening and data management will be supported using Covidence, and statistical analyses will be conducted using Python.

**Expected results:**

We anticipate including a substantial number of RCTs covering different BA subtypes. Based on previous literature, we expect beneficial intervention effects and potential gender-related response variations. Effect sizes may differ between men and women depending on addiction subtype and intervention characteristics.

**Discussion:**

If gender does not modify treatment effects, the results will support broad applicability; if differences emerge, they will guide tailored, gender-sensitive interventions. This may guide the adaptation of therapeutic approaches to better address the healthcare needs of different gender groups affected by BAs.

**Systematic review registration:**

PROSPERO CRD420251086582

**Supplementary Information:**

The online version contains supplementary material available at 10.1186/s13722-026-00668-0.

## Introduction

Behavioral addictions (BAs) are characterized by persistent or recurrent engagement (online and/or offline) in specific behaviors, accompanied by craving, impaired control over these behaviors (e.g., in terms of frequency or intensity), prioritization of the behavior to the extent that it results in functional impairment warranting treatment, and continuation or escalation despite negative consequences, in line with criteria outlined in the 11^th^ revision of the International Classification of Diseases (ICD-11) [1]. The World Health Organization (WHO) recognizes BAs under the category of disorders due to addictive behaviors (6C5). This category currently includes gambling disorder (6C50), gaming disorder (6C51), and further allows the diagnosis of additional behavioral patterns under other specified (6C5Y) or unspecified (6C5Z) disorders due to addictive behaviors [1]. Candidate phenomena for the latter category include buying-shopping disorder [2], internet-use disorder [3], social network-use and internet-communication disorders [4, 5], sexual behavior disorder [6], pornography-use disorder [7], and food addiction [8]. However, a variety of terms are used across the literature, reflecting the lack of universally accepted diagnostic criteria for many of these candidate conditions.

Reported prevalence rates of BAs vary substantially across subtypes. A comprehensive systematic review and meta-analysis by Alimoradi et al. [9], including 94 studies, reported prevalence estimates of food addiction (21.0%), social network-use disorder (15.1%), internet-use disorder (10.6%), sexual behavior disorder (9.4%), gambling and buying-shopping disorder (7.2% each), and gaming disorder (5.3%). Across all BA subtypes, the pooled global point prevalence was estimated at 11.1%. However, as many of the included studies were conducted during the COVID-19 pandemic, these estimates likely overstate prevalence and warrant cautious interpretation.

Complementing these findings, more recent large-scale meta-analyses provide more robust prevalence estimates for selected subtypes. For gambling disorder, Tran et al. [10] synthesized 380 representative samples covering 68 countries and more than 3.4 million individuals, estimating a global adult prevalence of 1.41% (approximately 80 million adults worldwide). Similarly, for digital BAs, Meng et al. [11] analyzed 2,127,059 participants across 64 countries and six WHO regions, reporting pooled prevalence estimates of social media addiction (17.4%), internet addiction (14.2%), cybersex addiction (8.2%), and gaming disorder (6.0%), with higher rates observed in lower-income countries and during the COVID-19 period.

Patterns of BAs seem to differ by gender: Men appear to be more frequently affected by gambling [10], gaming [12], internet use [13], and sexual behavior disorders [14], including pornography use disorder. Conversely, women seem to show higher prevalence rates of social network use disorder [12] and food addiction [15]. While gender differences in buying-shopping disorder have often been assumed, results from an unpublished meta-analysis currently under review suggest that prevalence rates may not differ between men and women [16, 17].

Beyond prevalence, BAs represent a substantial public health concern. Gambling disorder, for instance, is associated with elevated all-cause and suicide mortality compared with the general population and with individuals with other mental or somatic conditions [18, 19]. Contributing factors often include indebtedness and feelings of shame [20]. Although the incidence is presumed to be very low, non-violent deaths following excessively prolonged gaming sessions have been documented in a small number of case reports (e.g., Kuperczko et al. [21]), with reported cases predominantly involving men. Moreover, the harms of BAs extend beyond affected individuals, impacting families and the social environment, and generating societal costs such as unemployment, sick leave, reduced productivity, and increased healthcare expenditures [22].

Psychotherapeutic interventions such as cognitive behavioral therapy (CBT) have proven effective for the treatment of BAs [23], yet not all individuals benefit equally [24]. In substance use disorders, gender-sensitive and integrated therapeutic approaches have shown promise, particularly in addressing the needs of women with co-occurring mental health challenges [25]. For gambling disorder, initial evidence suggests gender-specific differences: men tend to achieve better short- and medium-term outcomes than women, though this advantage appears to diminish at follow-up [24]. For other disorders, such as sexual behavior disorder [26] and buying-shopping disorder [27], evidence on gender differences in treatment remains scarce. This is largely due to a lack of gender-sensitive study designs and insufficient gender-specific reporting, even when mixed-gender samples are included. To address this gap, systematic reviews and the collection of primary data are needed to enable an individual patient data meta-analysis (IPDMA) with a focused gender perspective.

Such evidence syntheses underpin evidence-based clinical practice guidelines. In Germany, a national S1 clinical practice guideline (i.e., a consensus-based guideline developed by expert agreement without a formal systematic evidence synthesis) for internet-related disorders is available, covering gaming, social-network use, buying-shopping, and pornography use [23]. Psychological interventions, particularly CBT, have been identified as first-line treatments [23], with promising evidence also supporting internet-based formats. However, it remains unclear whether such interventions are equally effective across genders in addressing BA-related psychopathology. A critical limitation lies in the evidence base itself. The S1 guideline emphasizes that most clinical trials - particularly in internet and gaming addiction - have predominantly recruited male participants. For example, the randomized controlled trial by Wölfling et al. [28] demonstrated high methodological rigor according to CONSORT criteria, but excluded female patients, thereby limiting its generalizability.

This imbalance is mirrored in the systematic reviews cited within the guideline [23, 27, 29–34]. While these reviews synthesize important findings on treatment outcomes, they rarely report sex- or gender-stratified results. An exception is the preregistered review on compulsive sexual behavior disorder [33], which explicitly underscored the need to address gender disparities in future treatment guidelines.

The guideline concludes that gender-sensitive investigations into the efficacy of psychological interventions are largely absent, representing a substantial research gap. This is particularly concerning given consistent evidence of gender differences in prevalence (e.g., Tran et al. [10] or Bőthe et al. [35]), clinical presentation (e.g., Mari et al. [36]), and comorbidities (e.g., de Mattos et al. [37]). Such disparities are also reflected in clinical trial populations, which may have unintentionally shaped treatments toward conditions more frequently observed in men (e.g., gambling) or in women (e.g., social-network use). Beyond prevalence, men and women with BAs often differ in motivational drivers, coping strategies, stress reactivity, and patterns of comorbidity, which may influence treatment engagement, adherence, and response [38–41]. Experimental and clinical research further indicates sex- and gender-related differences in affective processing, impulsivity, and emotion regulation, as well as in help-seeking behavior and symptom expression [42–44]. Although such differences are typically small at the population level [45, 46], they may become clinically relevant under conditions of high symptom severity, chronic stress exposure, or cumulative disadvantage [47].

Consequently, the lack of clarity about whether current interventions are equally effective across genders poses a challenge for clinicians striving to deliver equitable care. Rigorous, gender-based comparisons of intervention effects in BAs are therefore indispensable—not only to ensure fairness in health care delivery but also to guide the development and refinement of tailored, gender-sensitive treatment strategies.

Taken together, psychological interventions show promise for BAs, yet the evidence base rarely reports sex/gender-stratified outcomes and is often male-skewed, leaving the gender-specific effectiveness uncertain. To address this gap, we will conduct a systematic review and IPDMA of randomized trials to test whether gender modifies treatment effects on BA symptom severity at end-of-treatment and follow-up. This synthesis will provide the most precise estimates of gender-specific effectiveness to date and directly inform guideline recommendations and gender-sensitive care.

## Methods

### Eligibility criteria

Studies will be assessed for eligibility based on the criteria presented in Table 1.

**Table 1:** Inclusion and exclusion criteria

Domain	Inclusion	Exclusion
Study Characteristic	Must be reported in its most recent corrected version	Duplicate reports of the same trial, and studies that have been formally retracted
Population	Inclusion of both male and female patients	Studies including only one binary sex or gender group (women only/men only)
Study Design	RCT design with active (e.g., alternative interventions, treatment-as-usual) or passive (e.g., waitlist) control groups	Study types other than randomized controlled trials, including review articles, studies without a control group, and studies without randomization
Intervention Type	Psychological treatment studies examining manualized or non-manualized, therapist-led, internet-based, or self-help psychotherapeutic interventions for behavioral addicotions, including cognitive behavioral therapy, psychodynamic therapy, interpersonal therapy and other approaches (e.g., mindfulness-based interventions)	Trials investigating solely pharmacological treatments, without any psychological intervention
Target Disorder	Participants with a clinical behavioral addiction diagnosis according to DSM-IV, DSM-5, ICD-11 criteria, or clinically relevant behavioral addiction symptoms assessed via structured clinical interview (e.g., gambling disorder, gaming disorder, buying-shopping disorder, internet-use disorder, social network-use disorder, sexual behavior disorder, pornography-use disorder, food addiction)	Studies with non-clinical or subclinical cohorts (e.g., student samples, community samples) or with participants diagnosed with Parkinson’s disease or Parkinsonian symptoms
Medication		Studies involving participants currently receiving dopaminergic medication. This includes dopamine agonists: pramipexole, ropinirole, levodopa, rotigotine
Psychopathology Assessment	Assessment of behavioral addiction-related psychopathology using a validated questionnaire	
Time Points of Assessment	Baseline, end-of-treatment, and/or at least one follow-up assessment	
Outcome Measurement	Use of at least one validated primary or secondary outcome measure	
Primary Outcome	Assess symptom-severity of behavioral addiction as the primary outcome	
Secondary Outcome	Assess behavioral addiction associated psychopathology as the secondary outcome	
Secondary Outcome 2	Assess treatment-related factors as the secondary outcome	

There will be no restrictions on the year of publication or language; studies published in languages not spoken by the research team will be assessed for eligibility using automated translation tools. To avoid confounding, we will exclude trials conducted in Parkinson’s disease populations and trials in which participants are currently receiving dopaminergic medication. Parkinson’s disease is primarily associated with a depletion of nigrostriatal dopamine [48]. In addition, dopaminergic therapies, such as dopamine agonists, have been reported to induce impulse control disorders (e.g., Moore et al. [49]).

### Information sources

An electronic search will be conducted in accordance with the Peer Review of Electronic Search Strategies (PRESS) guideline. Databases to be searched include: Cochrane Database of Systematic Reviews, HTA/DARE, LILACS, Medline (via PubMed), PsycArticles, PsychINFO, PsycExtra, Catalogue of the German National Library of Medicine (via LIVIVO), PubPSYCH, Scopus, Web of Science. National and international trial registers will also be searched, including CENTRAL, ClinicalTrials.gov, Deutsches Register Klinischer Studien, EU Clinical Trials Register, U.K. Clinical Trials Gateway and the WHO International Clinical Trials Registry Platform.

Reference lists of all included articles will be manually screened to identify additional relevant studies not found through the database searches. Trials that are not yet completed will be documented but not included in the present IPDMA; they may be considered in future updates.

### Search strategy

Natural language terms and, where applicable, Medical Subject Headings (MeSH) related to relevant populations (e.g., buying-shopping disorder, gambling disorder, gaming disorder), interventions (e.g., CBT), and outcomes (e.g., BA symptom severity) will be used to construct broad search strings (see PROSPERO registration and Supplement for search string).

### Expected population

The expected population will comprise adolescents and adults participating in RCTs evaluating psychological interventions for BAs. Eligible populations will include (1) individuals diagnosed with BAs that are formally recognized in the ICD-11 (i.e., gambling disorder and gaming disorder), and (2) populations defined by clinically relevant BA symptoms assessed using structured clinical interviews, given the lack of universally accepted diagnostic criteria for some BAs (e.g., buying-shopping disorder, problematic social-network use, pornography-use disorder). To ensure transparency and comparability, the diagnostic approach and classification rationale used in each trial will be extracted in detail and considered in subgroup and sensitivity analyses. Where substantial heterogeneity in diagnostic definitions is present, this will be explicitly examined as a potential source of heterogeneity in the IPDMA.

### Expected interventions and control conditions

The review will include RCTs evaluating psychological interventions for BAs delivered in any format (e.g., individual or group-based, face-to-face or internet-based). Interventions are expected to primarily include CBT–based approaches, as well as other structured psychotherapeutic interventions (e.g., motivational interviewing, mindfulness-based or integrative approaches). Control conditions may include waitlist controls, treatment as usual, minimal or active psychological controls, or alternative psychotherapeutic interventions.

Given the heterogeneity across psychological interventions for BAs, intervention type will be coded in detail and considered in subgroup and moderator analyses (e.g., CBT-based vs. non-CBT-based interventions). Trials primarily evaluating pharmacological interventions will be excluded. Trials allowing concomitant pharmacological treatments will be included, if such treatments are balanced across intervention and control arms or explicitly reported as stable during the trial period; information on add-on pharmacological treatments will be extracted and considered in sensitivity analyses.

### Data management and selection process

All references will be managed using Covidence review software [50]. After automatic duplicate removal in Covidence, two investigators with relevant expertise (e.g., psychologists at the M.Sc. or PhD level) will independently screen titles, abstracts, and subsequently full texts. Full-text articles deemed potentially eligible will be assessed in detail by both reviewers according to the predefined eligibility criteria. Any disagreements will be resolved through discussion and, if necessary, by consultation with the first author (NML).

To enhance both efficiency and accuracy, we will implement an AI-assisted, recall-first co-screening workflow that uses large language models (LLMs) in a R programming environment (R version 4.5.0 [51]) as a further, non-authoritative reviewer at the title and abstract stage. Prior to deployment, the LLM will be selected and calibrated based on a gold-standard set of citations independently dual-screened by humans.

Prompts will encode the full inclusion/exclusion criteria and produce structured JavaScript Object Notation (JSON) outputs, a text data format, with a trichotomous decision (include/exclude/unsure), rationale, and tagged reasons for exclusion mapped to PRISMA categories. Selection of model will be made based on best mean classification out of three attempts after calibration and test major current deep-reasoning models (at the time of protocol writing: Gemini 2.5 pro, GPT-5, DeepSeek R1, Qwen3, gpt-oss, Magistral). If an open source model achieves the required threshold, such a model will be preferred to allow archiving for reproducibility.

Inference will be deterministic (temperature 0, top-p 1), with model family, version, and hash pinned and logged. Only bibliographic metadata, titles/abstracts, and indexing terms will be processed. Decision strategy: each record will be screened by two human reviewers, with the LLM operating in parallel as a non-authoritative reviewer. Any “include” or “unsure” from any party will advance the record to full-text review. Performance targets and safeguards: during calibration (Wilson 95% CI), the LLM should achieve true negative rate ≥ 95% with the lower confidence bound ≥ 90% against the human gold standard. If targets are not met, the system will revert to human-only screening until re-calibrated. Additional findings initially misclassified by human reviewers will be counted as true positives.

We will report human–human and human–LLM agreement (percent agreement, Cohen’s κ/Scott’s π), operating characteristics (recall, precision, estimated workload saved), and an audit trail (timestamps, prompts, model identifiers, decisions, rationales) sufficient for optimum reproducibility and PRISMA compliance. Full-text eligibility decisions and reasons for exclusion will be made only by humans. By leveraging LLMs in this semi-automated manner, we anticipate a substantial reduction in the time required for screening while maintaining high levels of accuracy and consistency [52–55].

Finally, the full texts of all selected articles will be reviewed independently by two reviewers to determine inclusion in the systematic review and IPDMA in accordance with the selection criteria. Any disagreements after full-text assessment will be resolved through discussion with, or arbitration by, the principal investigator (NML).

### Data extraction

Data extraction from published reports will be conducted independently by two reviewers using a standardized extraction form in Covidence [50]. Extracted information will include study characteristics, participant characteristics, intervention and control conditions, outcome measures, and reported results. Discrepancies between reviewers will be resolved through discussion and, if necessary, by consultation with the first author (NML). For trials contributing individual participant data, extracted information will be cross-checked against the shared datasets.

### Data collection process

The PIs of eligible RCTs will be invited to join the consortium (provisionally labelled “GENIOBA-IPDMA”) and share their data. If no response is received, reminders will be sent or alternative investigators from the same study will be contacted. Interested PIs will be asked to sign a data-sharing agreement and provide anonymized trial data. All datasets will be checked for completeness, plausibility, consistency with published results, and missing values. Data coding and transfer into the IPDMA database will be performed by supervised staff or trained team members. For each study, participant characteristics and screening results from the cleaned datasets will be cross-checked against the original datasets to identify discrepancies.

In cases of non-response from trial authors, the respective study cannot be included in the IPDMA. However, available aggregate data reported in the publication will be extracted and summarized descriptively as part of the systematic review.

### Data items

The following data will be requested and/or extracted: study title, reference, publication year, design and aim, participant information (e.g., diagnosis, comorbidities, gender, age, ethnicity), recruitment process, type of intervention, intervention setting, outcome measures, data for the primary and secondary endpoints, statistical methods, pre- to post measurement intervals, key results, authors’ conclusions, funding sources and conflicts of interest, as well as Risk-of-Bias (ROB). Additionally, where available, we will extract data on LGBTQ+^1^ participants, as these groups are underrepresented in clinical trials [56–59].

### Outcomes and prioritization

The primary outcome will be pre- to post-intervention (end-of-treatment) changes in BA symptom severity, assessed exclusively using standardized and validated disorder-specific instruments as defined by the respective trial authors. Instruments will be considered eligible, if they have been published in peer-reviewed literature with established reliability and validity. When multiple validated instruments are used within a study, outcomes will be harmonized using standardized effect sizes. Studies that do not employ validated instruments to assess BA symptom severity will be excluded at the full-text screening stage.

Secondary outcomes may include, where assessed using validated measures, changes in BA-related psychopathology (e.g., self-esteem, obsessive-compulsive traits, depressive symptoms, impulsivity), comorbid substance-use disorders, executive functioning deficits, and treatment-related aspects (e.g., dropout rates, remission). Secondary outcomes will be synthesized where assessed using validated measures.

### Risk-of Bias in individual studies

Two reviewers with methodological expertise (e.g., a psychologist at the M.Sc. or PhD level) will independently assess the ROB using the Cochrane revised ROB 2 tool for RCTs.

### Data synthesis

This systematic review with IPDMA will investigate the outcomes of psychotherapeutic interventions for improving psychopathology in men and women with BAs. First, we will conduct a systematic narrative synthesis of reported gender differences, summarizing key themes (e.g., intervention types, diagnostic subgroups) based on lists of themes and subthemes identified independently by two reviewers. Second, for each primary and secondary outcome measure, we will conduct an IPDMA of treatment × gender interactions [60, 61], the recommended approach for assessing variation in treatment effects across patient subgroups. We chose a two-stage approach to enable the inclusion of RCTs that are unwilling to share individual patient data but can provide the required interaction estimates.

In the first stage of the IPDMA, linear regression models with restricted maximum likelihood (REML) estimation will be fitted to assess treatment (intervention vs. control) × gender (men vs. women) interactions on post-treatment outcomes, using an intention-to-treat approach. Models will include treatment, gender, and their interaction, and will adjust for trial-specific prognostic factors such as baseline scores (analysis of covariance approach), study center (for multi-center trials), and patient age.

In the second stage, within-trial interaction estimates will be combined using an inverse-variance weighted, random-effects meta-analysis with REML estimation, allowing for between-study heterogeneity. Exploratory trial-level moderators of the treatment × gender interaction (e.g., diagnosis group, setting such as inpatient vs. outpatient or group vs. individual therapy), and treatment approach such as CBT will be examined via meta-regression. A two-tailed α < 0.05 will be applied for significance testing.

Results of the IPDMA will be reported as forest plots for the different interventions and BA diagnoses (e.g., gambling disorder, gaming disorder, buying-shopping disorder). Statistical heterogeneity will be quantified using the I^2^ statistic, with I^2^ ≥ 50% indicating moderate and I^2^ ≥ 75% high heterogeneity. Heterogeneity will be addressed at multiple levels. Clinical heterogeneity arising from differences in diagnostic definitions (e.g., ICD-11–defined disorders vs. symptom-defined BAs) will be documented in detail and examined through subgroup and moderator analyses. Intervention-related heterogeneity (e.g., CBT-based vs. non-CBT-based approaches, delivery format, intensity) and differences in control conditions (e.g., waitlist, treatment as usual, active controls) will likewise be coded a priori and explored as trial-level moderators within meta-regression models. Where substantial heterogeneity is observed, sensitivity analyses will be conducted to assess the robustness of findings by excluding specific diagnostic groups, intervention types, or control conditions.

To evaluate whether the two-staged IPDMA is sufficiently powered to detect gender differences, we conducted an a priori, simulation-based power analysis based on the methodology by Kovalchik and Cumberland [40]. This approach requires summary data on intervention outcomes, gender distribution, and group sizes, which were available from several published RCTs. The simulation indicated that a subset of 11 studies would provide 75% power to detect a medium-sized interaction effect (e.g., a gender difference in treatment-induced symptom changes of 0.5 SD).

Separate quantitative syntheses for specific intervention types or diagnostic subgroups will be conducted only where sufficient data are available. Where subgroup-specific analyses would be underpowered, intervention type and diagnostic category will be examined as moderators within the overall IPDMA framework to preserve statistical power for detecting gender-related effects.

### Meta-bias(es)

Publication biases will be assessed using contour-enhanced funnel plots, with interaction effects on the horizontal axis and standard errors (SEs) on the vertical axis, supplemented by statistical tests of funnel plot asymmetry. Regression-based sensitivity analyses will be conducted to evaluate the robustness of results by excluding trials rated as high ROB. As an additional sensitivity analysis, results of the two-stage IPDMA will be compared with those from a one-stage approach using a generalized linear model with trial-stratified parameters for all effects outside the interaction term [39].

### Confidence in cumulative evidence

The overall quality of evidence for the primary outcomes will be independently rated by two reviewers according to the GRADE framework. The assessment will consider random sequence generation, allocation concealment, outcome completeness, and completeness of reporting. Ratings of both reviewers will be compared, with disagreements resolved through consultation with the first author (NML). Quality and ROB ratings will also be included as moderators in the analysis.

## Discussion

Several limitations should be acknowledged. First, the feasibility of conducting an IPDMA depends on the willingness of PIs to share individual participant data; non-response or refusal may reduce the number of RCTs contributing to the IPDMA. To mitigate this risk, initial commitments to data sharing by PIs have already been sought for, and an alternative approach will be offered, in which investigators may run a standardized analysis script locally and share the resulting outputs only. This strategy is supported by the study team’s established research network in the field of BAs.

Second, the review will be restricted to published RCTs. The exclusion of grey literature may introduce publication bias and potentially overestimate treatment effects; this will be considered when interpreting the findings and formally assessed where feasible.

Third, diagnostic heterogeneity represents an inherent challenge, as some BAs are formally defined in the ICD-11 whereas others lack universally accepted diagnostic criteria. Differences in diagnostic definitions and assessment approaches will therefore be documented and examined as potential sources of heterogeneity.

Finally, a limitation concerns the distinction between sex assigned at birth and gender. Many RCTs rely on binary sex categories without explicitly distinguishing between sex and gender identity [59]. Consequently, analyses will primarily reflect groupings, which may not fully capture gender diversity or gender-related mechanisms; where available, additional gender identity information will be reported descriptively.

Despite these limitations, the IPDMA approach is considered the gold standard of evidence synthesis, as it offers important advantages over conventional aggregate data meta-analyses [62, 63]. By using individual participant data, IPDMA allows for harmonization of outcomes and covariates across trials, adjustment for baseline characteristics at the participant level, and more reliable intention-to-treat analyses, thereby reducing bias associated with aggregate analyses. Importantly, IPDMA enables statistically valid and adequately powered subgroup analyses, such as the investigation of treatment × gender interactions, which are often unreliable or infeasible in standard meta-analyses based solely on published summary data. Moreover, IPDMA facilitates a more detailed and transparent exploration of heterogeneity across populations, interventions, and study designs.

Despite the growing recognition of BAs, comprehensive research on therapeutic modalities and their effectiveness remains urgently needed. Such research is essential to clarify how psychological interventions improve outcomes for individuals with BAs, particularly with respect to gender-related disparities. However, this aspect is still insufficiently addressed in the literature, and no systematic review on treatment outcomes in clinical effectiveness trials has yet applied an IPDMA framework.

The planned IPDMA will therefore provide robust evidence either for gender-related differences in intervention effects – informing tailored recommendations and guiding improvements in treatment modalities – or for equivalence, thereby reassuring clinicians about the applicability of current therapeutic approaches across genders. In doing so, it will support the optimizing of health care delivery and highlight areas where further research and refinement are required.

## Electronic supplementary material

Below is the link to the electronic supplementary material.

Supplementary material 1

## Data Availability

Meta-analysis coding sheets, coded data, codebooks, analysis syntax as well as any other non-confidential documents created as part of the systematic review will be made publicly available using the Open Science Framework (https://osf.io/) repository, adhering to FAIR principles. Data sharing is not applicable to this protocol as no datasets were generated or analyzed.

## Abbreviations

BA: Behavioral Addiction
CBT: Cognitive Behavioral Therapy
CONSORT: Consolidated Standards of Reporting Trials
DSM: Diagnostic and Statistical Manual of Mental Disorders
FAIR: Findability, Accessibility, Interoperability, and Reuse of digital assets
GRADE: Grading of Recommendations Assessment, Development, and Evaluation
ICD: International statistical Classification of Diseases and related health problems
IPDMA: Individual Patient Data Meta-Analysis
LLM: Large Language Model
MeSH: Medical Subject Headings
PRESS: Peer Review of Electronic Search Strategies
RCT: Randomized Controlled Trial
RoB: Risk-of-Bias

## Footnotes

1 In this article, LGBTQ+ refers to a spectrum of sexuality and gender identities, including (but not limited to) lesbian, gay, bisexual, transgender, queer/questioning, and other identities not explicitly mentioned in the acronym.
