# Supplementary material for: Addressing gender disparities in the efficacy of psychological interventions for behavioral addictions: protocol for a systematic review and individual patient data meta-analysis of randomized controlled trials
Source: Addict Sci Clin Pract. 2026 May 22;21:48. doi: 10.1186/s13722-026-00668-0 (PMC13231642; doi:10.1186/s13722-026-00668-0)
Supplement: Supplementary file 1 — Supplementary material 1 [file 13722_2026_668_MOESM1_ESM.docx]

# Addressing Gender Disparities in the Efficacy of Psychological Interventions for Behavioral Addictions: Protocol for a Systematic Review and Individual Patient Data Meta-Analysis of Randomized Controlled Trials

# Supplement

**Search String for PubMed**

(((porn addict*[Title/Abstract]) OR (porn-addict*[Title/Abstract]) OR (pornography addict*[Title/Abstract]) OR (pornography-addict*[Title/Abstract]) OR (addictive porn*[Title/Abstract]) OR (cybersex addict*[Title/Abstract]) OR (cybersex-addict*[Title/Abstract]) OR (addictive cybersex*[Title/Abstract]) OR (sexual addict*[Title/Abstract]) OR (sexual-addict*[Title/Abstract]) OR (addictive sex*[Title/Abstract]) OR (problematic porn*[Title/Abstract]) OR (problematic sex*[Title/Abstract]) OR (problematic cybersex*[Title/Abstract]) OR (hypersex*[Title/Abstract]) OR (compulsive sex*[Title/Abstract]) OR (compulsive porn*[Title/Abstract]) OR (compulsive cybersex*[Title/Abstract]) OR (sexual compuls*[Title/Abstract]) OR (impulsive sex*[Title/Abstract]) OR (impulsive porn*[Title/Abstract]) OR (impulsive cybersex*[Title/Abstract]) OR (sexual impuls*[Title/Abstract]) OR (obsessive sex*[Title/Abstract]) OR (obsessive porn*[Title/Abstract]) OR (obsessive cybersex*[Title/Abstract]) OR (sexual obsess*[Title/Abstract]) OR (sexual preoccupation[Title/Abstract]) OR (sexual hyperactivity[Title/Abstract]) OR (out of control sexual[Title/Abstract]) OR (out-of-control-sexual[Title/Abstract]) OR (paraphilia related[Title/Abstract]) OR (non-paraphilic[Title/Abstract]) OR (sexual behavior disorder[Title/Abstract]) OR (sexual-behavior-disorder[Title/Abstract]) OR (pornography-use disorder[Title/Abstract]) OR (pornography use disorder[Title/Abstract]) OR (pornography-use-disorder[Title/Abstract])

OR (buying addict*[Title/Abstract]) OR (buying-addict*[Title/Abstract]) OR (addictive buyi*[Title/Abstract]) OR (compulsive buyi*[Title/Abstract]) OR (impulsive buyi*[Title/Abstract]) OR (problematic buyi*[Title/Abstract]) OR (pathological buyi*[Title/Abstract]) OR (excessive buyi*[Title/Abstract]) OR (compensatory buyi*[Title/Abstract]) OR (obsessive buyi*[Title/Abstract]) OR (addictive buye*[Title/Abstract]) OR (compulsive buye*[Title/Abstract]) OR (impulsive buye*[Title/Abstract]) OR (problematic buye*[Title/Abstract]) OR (pathological buye*[Title/Abstract]) OR (excessive buye*[Title/Abstract]) OR (compensatory buye*[Title/Abstract]) OR (obsessive buye*[Title/Abstract]) OR (shopping addict*[Title/Abstract]) OR (shopping-addict*[Title/Abstract]) OR (addictive shop*[Title/Abstract]) OR (compulsive shop*[Title/Abstract]) OR (impulsive shop*[Title/Abstract]) OR (problematic shop*[Title/Abstract]) OR (pathological shop*[Title/Abstract]) OR (excessive shop*[Title/Abstract]) OR (compensatory shop*[Title/Abstract]) OR (obsessive shop*[Title/Abstract]) OR (shopping problem*[Title/Abstract]) OR (spending addict*[Title/Abstract]) OR (spending-addict*[Title/Abstract]) OR (addictive spend*[Title/Abstract]) OR (compulsive spend*[Title/Abstract]) OR (impulsive spend*[Title/Abstract]) OR (problematic spend*[Title/Abstract]) OR (pathological spend*[Title/Abstract]) OR (excessive spend*[Title/Abstract]) OR (compensatory spend*[Title/Abstract]) OR (obsessive spend*[Title/Abstract]) OR (purchasing addict*[Title/Abstract]) OR (purchasing-addict*[Title/Abstract]) OR (addictive purchas*[Title/Abstract]) OR (compulsive purchas*[Title/Abstract]) OR (impulsive purchas*[Title/Abstract]) OR (problematic purchas*[Title/Abstract]) OR (pathological purchas*[Title/Abstract]) OR (excessive purchas*[Title/Abstract]) OR (compensatory purchas*[Title/Abstract]) OR (obsessive purchas*[Title/Abstract]) OR (onioman*[Title/Abstract]) OR (overshop*[Title/Abstract]) OR (hyperspend*[Title/Abstract]) OR (overspend*[Title/Abstract]) OR (buying shopping disorder[Title/Abstract]) OR (buying-shopping disorder[Title/Abstract]) OR (buying-shopping-disorder[Title/Abstract]) OR (buying shopping-disorder[Title/Abstract]) OR (buying disorder[Title/Abstract]) OR (buying-disorder[Title/Abstract]) OR (shopping-disorder[Title/Abstract]) OR (shopping disorder[Title/Abstract])

OR (gambling addict*[Title/Abstract]) OR (gambling-addict*[Title/Abstract]) OR (compulsive gambling[Title/Abstract]) OR (problematic gambling[Title/Abstract]) OR (gambling disorder[Title/Abstract]) OR (gambling-disorder[Title/Abstract]) OR (pathological gambling[Title/Abstract]) OR (excessive gambling[Title/Abstract]) OR (impulsive gambling[Title/Abstract])

OR (problematic gaming[Title/Abstract]) OR (problematic online gaming[Title/Abstract]) OR (problematic video gaming[Title/Abstract]) OR (problematic internet gaming[Title/Abstract]) OR (gaming disorder[Title/Abstract]) OR (gaming-disorder[Title/Abstract]) OR (pathological gaming[Title/Abstract]) OR (pathological online gaming[Title/Abstract]) OR (pathological video gaming[Title/Abstract]) OR (pathological internet gaming[Title/Abstract]) OR (compulsive gaming[Title/Abstract]) OR (compulsive online gaming[Title/Abstract]) OR (compulsive video gaming[Title/Abstract]) OR (compulsive internet gaming[Title/Abstract]) OR (excessive gaming[Title/Abstract]) OR (excessive online gaming[Title/Abstract]) OR (excessive video gaming[Title/Abstract]) OR (excessive internet gaming[Title/Abstract]) OR (addictive gaming[Title/Abstract]) OR (addictive online gaming[Title/Abstract]) OR (addictive video gaming[Title/Abstract]) OR (addictive internet gaming[Title/Abstract]) OR (video gaming[Title/Abstract]) OR (online gaming[Title/Abstract]) OR (internet gaming[Title/Abstract])

OR (social media addict*[Title/Abstract]) OR (social-media addict*[Title/Abstract]) OR (social media-addict*[Title/Abstract]) OR (social-media-addict*[Title/Abstract]) OR (addictive social media[Title/Abstract]) OR (addictive social-media[Title/Abstract]) OR (problematic social media[Title/Abstract]) OR (problematic social-media[Title/Abstract]) OR (compulsive social media[Title/Abstract]) OR (compulsive social-media[Title/Abstract]) OR (excessive social media[Title/Abstract]) OR (excessive social-media[Title/Abstract]) OR (social network addict*[Title/Abstract]) OR (social-network addict*[Title/Abstract]) OR (social network-addict*[Title/Abstract]) OR (social-network-addict*[Title/Abstract]) OR (addictive social network[Title/Abstract]) OR (addictive social-network[Title/Abstract]) OR (problematic social network[Title/Abstract]) OR (problematic social-network[Title/Abstract]) OR (compulsive social network[Title/Abstract]) OR (compulsive social-network[Title/Abstract]) OR (excessive social network[Title/Abstract]) OR (excessive social-network[Title/Abstract]) OR (social media use disorder[Title/Abstract]) OR (social-media use disorder[Title/Abstract]) OR (social-media-use-disorder[Title/Abstract]) OR (social-network use disorder[Title/Abstract]) OR (social network use disorder[Title/Abstract]) OR (social-network-use-disorder[Title/Abstract]) OR (problematic social media use[Title/Abstract]) OR (problematic social-media use[Title/Abstract]) OR (problematic social-network use[Title/Abstract]) OR (problematic social network use[Title/Abstract]) OR (addiction to social media[Title/Abstract]) OR (addiction to social network*[Title/Abstract]) OR (addiction to social-media[Title/Abstract]) OR (addiction to social-network*[Title/Abstract]) OR (social network use disorder[Title/Abstract]) OR (social-network use disorder[Title/Abstract]) OR (social-network-use-disorder[Title/Abstract]) OR (internet-use disorder[Title/Abstract]) OR (internet-use-disorder[Title/Abstract]) OR (internet use disorder[Title/Abstract]) OR (excessive internet use[Title/Abstract]) OR (addictive internet use[Title/Abstract]) OR (problematic internet use[Title/Abstract]) OR (problematic TikTok use[Title/Abstract]) OR (problematic TikTok-use[Title/Abstract]) OR (addictive TikTok use[Title/Abstract]) OR (addictive TikTok-use[Title/Abstract]) OR (excessive TikTok use[Title/Abstract]) OR (excessive TikTok-use[Title/Abstract]) OR (addiction to TikTok[Title/Abstract]) OR (TikTok addiction[Title/Abstract]) OR (addictive Instagram use[Title/Abstract]) OR

(addictive Instagram-use[Title/Abstract]) OR (problematic Instagram use[Title/Abstract]) OR (problematic Instagram-use[Title/Abstract]) OR (excessive Instagram use[Title/Abstract]) OR (excessive Instagram-use[Title/Abstract]) OR (addiction to Instagram[Title/Abstract]) OR (Instagram addiction[Title/Abstract]) OR (problematic Facebook use[Title/Abstract]) OR (problematic Facebook-use[Title/Abstract]) OR (addictive Facebook use[Title/Abstract]) OR (addictive Facebook-use[Title/Abstract]) OR (excessive Facebook use[Title/Abstract]) OR (excessive Facebook-use[Title/Abstract]) OR (addiction to Facebook[Title/Abstract]) OR (Facebook addiction[Title/Abstract])

OR (food addict*[Title/Abstract]) OR (addictive eating[Title/Abstract]) OR (food addiction[Title/Abstract]) OR (eating addiction[Title/Abstract]))

AND ((intervention*[Title/Abstract]) OR (treatment*[Title/Abstract]) OR (therap*[Title/Abstract]) OR (psychological intervention[Title/Abstract]) OR (psychotherapy*[Title/Abstract]) OR (cognitive behavioral therapy[Title/Abstract]) OR (CBT[Title/Abstract]) OR (internet-based therap*[Title/Abstract]) OR (internet-based intervention) OR (internet based intervention) OR (mindfulness[Title/Abstract]) OR (dialectical behaviour therapy[Title/Abstract]) OR (DBT[Title/Abstract]) OR (mentalization*[Title/Abstract]) OR (interpersonal psychotherapy*[Title/Abstract]) OR (psychotherap*[Title/Abstract]) OR (counsel*[Title/Abstract]) OR (self-help group*[Title/Abstract]) OR (self help groups[Title/Abstract]) OR (self-help-group*[Title/Abstract]) OR (mental health services[Title/Abstract]) OR (psychosocial intervention*[Title/Abstract]) OR (program*[Title/Abstract]) OR (cognitive behavio*[Title/Abstract]) OR (behavir therap*[Title/Abstract]) OR (acceptance[Title/Abstract]) OR (psychodynamic[Title/Abstract]) OR (interpersonal[Title/Abstract]) OR (compassion[Title/Abstract]) OR (family therap*[Title/Abstract]) OR (family-based*[Title/Abstract]) OR (self-help[Title/Abstract]) OR (treatment outcome*[Title/Abstract]) OR (patient outcome assessment[Title/Abstract]) OR (psychopathology[Title/Abstract]) OR (symptom severit*[Title/Abstract]) OR (motivational interview*[Title/Abstract]) OR (psychoeducation*[Title/Abstract])))
